# Resistance to demineralisation of adjacent enamel and dentine, fluoride release and dentine bond strength of fluoride-containing self-etch adhesive systems

**DOI:** 10.4317/jced.56170

**Published:** 2020-04-01

**Authors:** Maryam Pirmoradian, Shima Esmailzadeh, Sotoudeh Davaie, Bahaa-Abdulrazzaq-Jerri Albakhakh, Bahareh Sanaee, Elnaz Asgari, Kiana Shekofteh, Sareh Habibzadeh, Marjan Behroozibakhsh

**Affiliations:** 1 DDS, Ph.D candidate Department of Dental Biomaterials, School of Dentistry, Tehran University of Medical Sciences, Tehran, Iran; 2 DDS, Ph.D candidate Research Center for Science and Technology in Medicine, Tehran University of Medical Sciences, Tehran, Iran; 3 DDS, Ph.D candidate Department of Dental Biomaterials, School of Dentistry, International Campus, Tehran University of Medical Sciences, Tehran, Iran; 4 DDS, MSc, Department of Prosthodontics, School of Dentistry, Tehran University of Medical Sciences, International campus, Tehran, Iran

## Abstract

**Background:**

The current study aimed to assess the amount of fluoride released from fluoride-containing dental adhesives and its effect on micro-tensile bond strength (µTBS) and on resistance to demineralisation of dentine and enamel.

**Material and Methods:**

Two fluoride-containing dental adhesives, and a fluoride-free adhesive were used as experimental adhesives. After thermal cycling the µ-TBS of adhesives to dentine and the failure mode were assessed. The fluoride release and cross-sectional microhardness (CSMH) of specimens were measured before and after one day, 7 and 28 days of pH-cycling. The data were analysed using one-way ANOVA, Weibull statistics and repeated measures ANOVA.

**Results:**

The results indicated a significant difference between the group of FL and both the SE and LBF groups (*p*≤0.001). The CSMH values of both the dentine and enamel underneath the adhesives was reduced at 28 th day of the pH-cycling compared to the baseline (*p*≤0.001). From day 1 to day 28, the released fluoride declined in both the fluoride containing dental adhesives (*p*≤0.001).

**Conclusions:**

Based on the results, the released fluoride from dental adhesives may adversely influence the bond strength and durability of the resin/dentine interface. Moreover, the released fluoride didn’t improve the resistance to demineralisation of adjacent enamel and dentine to bond interface.

** Key words:**Fluoride release, micro-tensile bond strength, microhardness, fluoride-containing adhesives.

## Introduction

Secondary caries is one of the main causes of resin composites failures. Such failures may be due to an improper marginal seal at the interface of tooth structure and restorative material or demineralisation of adjacent enamel and dentine to dental adhesives (1). It has been reported that the fluoride-releasing properties of some dental materials can prevent secondary caries formation. The anticariogenic effect of fluoride is well documented. A variety of mechanisms have been described to explain the anticariogenic effects of fluoride, including the reduction of demineralisation, enhancement of remineralisation, interference with plaque formation and inhibition of microbial activity (2,3). Accordingly several researchers added fluoride fillers with different sources to dental restorative materials to inhibit secondary caries, and just now a variety of fluoride-containing dental materials including glass-ionomers, hybrid-ionomers, resin composites and dental adhesives are available in the market (4). It seems that enhancement of remineralisation is the most likely mechanism in this regard. Controversial results have been reported concerning the anticariogenic effect of fluoride-containing dental adhesives and composites. In an attempt Vasconcelos *et al.* (5) resulted that all adhesive systems were unable to inhibit secondary caries formation regardless of fluoride content (5). Pellizzari *et al.* (6) reported that the fluoride ion release of self-adhesive resin cements and their effect on inhibition from demineralisation of tooth structures was lower than that of glass ionomer luting cements (6). Recent studies have shown that fluoride containing bioglasses can improve the bioactivity of dental adhesives. They have expressed that these novel adhesives may discourage the occurrence and progression of early caries lesions (7,8). The cariostatic effect of fluoride containing dental materials is attributed to the amount of fluoride released from the materials as well as the ability of fluoride to incorporate into the adjacent tooth structures (9). The composition, concentration, source and size of fluoride containing filler particles, along with the permeability and solubility of the resin matrix of dental resin materials, can affect the fluoride releasing property (6,10). Some compositional and experimental factors including the composition, pH of saliva, mixing procedure, powder–liquid ratio of two-phase-systems, exposed area, curing time, and type of storage media also can affect the fluoride release of materials (4). Incorporation of water-soluble salts like NaF or SnF2 for the development of fluoride-releasing resins can increase the fluoride release of the material. However, enhancement of fluoride release may lead to some voids throughout the resin matrix as the result of fluoride leaching out of the matrix (4). Nevertheless, several researchers have indicated that the fluoride content can improve the bond strength of fluoride-containing dental resins to tooth mineral structure (11,12).

The aim of the current study was to assess the fluoride release, and dentine bond strength of self-etch fluoride-containing dental adhesives. Moreover. we aimed to evaluate the resistance to demineralisation of the dentine and enamel adjacent to the fluoridated dental adhesives.

The null hypotheses of the study were: (1) The amount of fluoride ions released from fluoride-containing self-etch adhesives would be similar during pH-cycling (2) Released fluoride would not influence the bond strength of fluoridated adhesives to dentine; and (3) Released fluoride would not affect the resistance to demineralisation of dentine and enamel adjacent to the bond interface.

## Material and Methods

Non-carious human third molars, extracted for medial indications were chosen for this study. The extracted teeth were cleaned and stored in 0.5% chloramine solution for a week. They were observed under a stereomicroscope (EZ4D; Leica Microsystems Ltd., Singapore) and the damaged tooth was excluded. The teeth then were randomly divided to different groups for different test methods.

- Microtensile bond strength test

The selected teeth were divided to three groups (n=5) according the adhesive system used. The occlusal surfaces of teeth and the teeth roots were removed using a diamond saw (Isomet, Buehler, Lake Bluff, II, USA). The surfaces of exposed dentine were observed with a stereomicroscope (EZ4D; Leica Microsystems Ltd., Singapore) for evaluation of any pulp horns and for inspection of any remaining enamel. Afterwards using 600 grit silicon carbide paper a standardized smear layer was created on the prepared dentine surfaces.

The adhesive systems including a fluoride free self-etch adhesive Clearfil SE Bond (Kuraray, Medical Inc, Tokyo, Japan) and two fluoride-containing self-etch adhesive, Fluorobond II (Shofu Inc., Kyoto, Japan) and Clearfil Liner Bond F(Kuraray Noritake Dental Inc., Okayama, Japan), were applied on the exposed dentine surfaces according to the manufacturer’s instructions. A 6-mm-thick build-up of composite resin (Shade A3, Adper Scotchbond Multi-Purpose, 3M ESPE, St. Paul,MN,USA ) was incrementally (in 2-mm-thick layers) light polymerized using an LED light-curing unit (Demi, Kerr, USA) with an output of over 1200 mW/cm2 for 20 s. The manufacturers, compositions, lot numbers and manufacturer’s instructions of the materials used in this study are listed in Table 1.

**Table 1:**
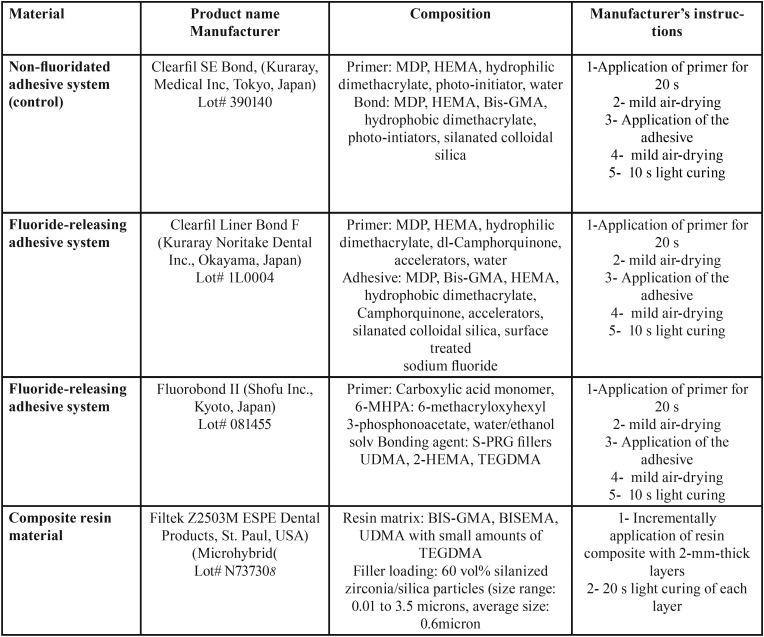
Commercial name, composition and manufacturer’s instructions of materials used in this study.

The prepared samples were then stored in artificial saliva at 37°C for 24 hours. Thereafter the teeth were sectioned serially parallel to long axis of teeth in mesio-distal and bucco-lingual directions at 1-mm intervals using a water-cooled diamond saw (Isomet, Buehler, Lake Bluff, II, USA). At least six 1 × 1× 12-mm2 longitudinal rectangular sticks with two halves of resin composite and dentine were obtained from each tooth. The beams were then subjected to 3000 thermal cycles between 5°C and 55°C with a dwell time of 20 s.

The beams then were fixed in a microtensile testing apparatus with a cyanoacrylate adhesive and subjected to microtensile testing using a universal testing machine with a 6.0-kg load cell (Bongshin®, Bongshin Loadcell Co, LTD, Seoul, Korea) at a crosshead speed of 1mm/min. ‘Pre-testing failures’, including debonded samples during the thermal cycling were not considered in the statistical analysis (13).

The fractured sticks of debonded specimens were gold coated and observed under scanning electronmicroscopy (Stereoscan S 360 Cambridge, Japan) to determine the mode of failure. Failure modes were classified into four groups described below.

1) Adhesive at the dentine-resin composite interface 2) cohesive failure in dentine 3) cohesive failure in resin composite 4) mixed failure.

- Cross-sectional microhardness test

For a microhardness test, 39 healthy non carious teeth were randomly divided into three groups according to adhesive system.

2 mm depth, 3 mm occlusogingival high and 4 mm mesiodistal width Class V cavities were prepared using a flat-ended cylindrical bur and a high speed equipment under water cooling on the buccal or lingual surfaces of the human third molars. The occlusal and cervical margins were located in enamel and dentine respectively. After preparation of five teeth the bur was replaced with a new one to maintain the uniformity of cavities.

The adhesive systems were applied according to the manufacturer’s instructions on the surfaces of prepared cavities. The composite resin (Shade A3, Adper Scotchbond Multi-Purpose, 3M ESPE, St. Paul,MN,USA ) was then incrementally placed (1-mm-thick layers) to the cavities and light polymerized using an LED light-curing unit (Demi, Kerr, USA) for 20s.

Afterwards the teeth with their restored cavities were sectioned mesiodistally into four slabs using a diamond saw (Isomet, Buehler, Lake Bluff, II, USA). The slabs were assigned into the four groups according to the days of pH-cycling process: 0 day, 1 day, 7 days and 28 days. In vitro pH-cycling scheme was used for 156 obtained slabs including 18 h in remineralisation solution and 6 h in demineralisation solution on each day (14). The demineralising solution contained CaCl2 12mM, NaH2PO4 2.2 mM, NaCl 100 mM, Acetic acid 50 mM and the pH of solution was adjusted at 4.5 using NaOH. The remineralising solutions at pH 7 composed of CaCl2 1.0 mM, NaH2PO4 3.0 mM, NaCl 100 mM.(15) Between two cycles, the enamel slabs were individually washed and then dried.

The enamel slabs then were embedded in polymethyl methacrylate, and hand-polished using silicon carbide papers up to 2,000 grit to produce a flat surface. A cross-sectional microhardness (CSMH) using a microhardness tester (V-Test II, Baresiss, Germany) with a Vickers diamond indenter was carried out to evaluate the demineralisation of enamel and dentine adjacent to adhesive resin. The microhardness measurement of enamel slabs was done prior to the pH-cycling procedure, one day after pH-cycling, seven days after pH-cycling and 28 days after pH-cycling. Six Vickers Pyramid Numbers (HV) from each sample were recorded under a load of 200 g and 50 gr for 15 s in enamel and dentine respectively.

- Fluoride release

The De/Re solutions of individually pH-cycled specimens of Clearfil Liner Bond F (Kuraray Noritake Dental Inc., Okayama, Japan) and Fluorobond II (Shofu Inc., Kyoto, Japan) adhesive systems after a day, 7 days and 28 days of pH-cycling procedure were collected for fluoride release measurement.

**Figure 1:**
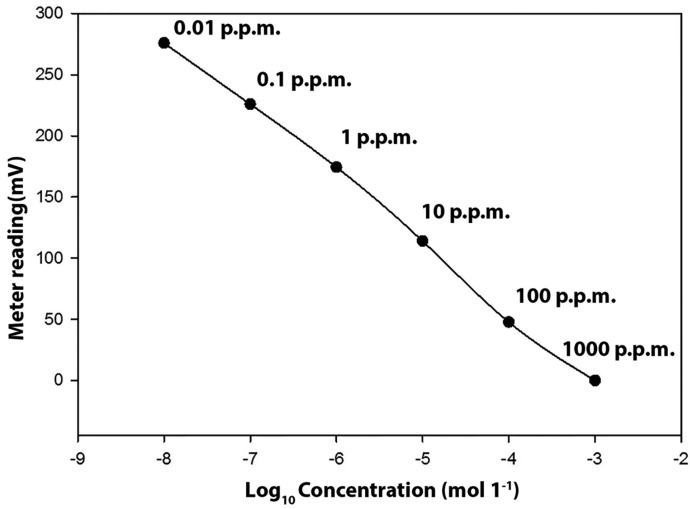
Typical calibration plots of of mV reading against known concentrations of fluoride.

The fluoride concentration of solutions was determined using the ion-selective electrode (ISE) method. A calibration procedure first was performed. A 100 p.p.m. standard solution of sodium fluoride (98.5%, Merck company) with deionized water was prepared and stored in a clean plastic container. A series of 12 x dilutions including solutions of 100, 50, 25,10, 5, 2.5, 1, 0.5, 0.05, and 0.01 then were produced and the calibration graph plotted (Fig. 1).

For the analysis of fluoride ion release, 2 ml samples of De/Re solutions were mixed with 2 ml of freshly TISAB lll solution and stirred. To prepare the TISAB lll solution 17.65 g CDTA (1,2-cyclohexanediaminetetraacetic) (Merck company) was added to 500 ml distilled water and 40 % sodium hydroxide also was added drop by drop to dissolve the salt. Then 300 g sodium citrate dehydrate and 60 g sodium chloride were mixed with the solution under stirring. The final volume of solution was adjusted to one liter by adding distilled water.

The fluoride concentration of solutions was measured using a pH/Ion meter (781 pH/Ion meter, Metrohm, Switzerland) attached to an ion selective electrode for fluoride (number 6.0502.150, Metrohm)) and a graph of the meter reading vs. known concentration of fluoride was plotted.

- Statistical analysis

After confirmation of normal distribution of data, the one-way analysis of variance (ANOVA) followed by a post-hoc Tukey’s test was performed to determine the micotensile bond strength of three different adhesive systems. A repeated measures ANOVA followed by a Bonferroni post hoc test was used to determine the CSMH and fluoride release of samples before pH-cycling and a day, 7 days and 28 days of pH-cycling process. All statistical analyses were performed at significance level of 0.05 using SPSS 23.0 for Windows.

The bond strength data were also statistically analysed using Weibull analysis (Weibull++6; ReliaSoft, Tucson, AZ, USA). The Weibull modulus and also characteristic strength (63.2% unreliability) were calculated by the estimation of Rank Regression on X (RRX) and Fisher Matrix Confidence Bounds (FM). The 10% and 90% unreliability levels of the specimens of three adhesives also compared by ordering the bond strength values from the lowest to the highest value and using the following equation: Pf=(i-0.5)/n

where i is the i th datum and n is the total number of data points of each group

## Results

- Microtensile bond strength

The mean microtensile bond strength values, standard deviation, and Weibull parameters of a fluoride-free and two fluoride-containing self-etch adhesives are presented in Table 2. One-way ANOVA showed no statistically significant difference between the groups of SE and LBF adhesives (*p*=0.28). However, the results indicated statistically significant difference between the group of FL and both SE and LBF groups (p≤0.001).

**Figure 2:**
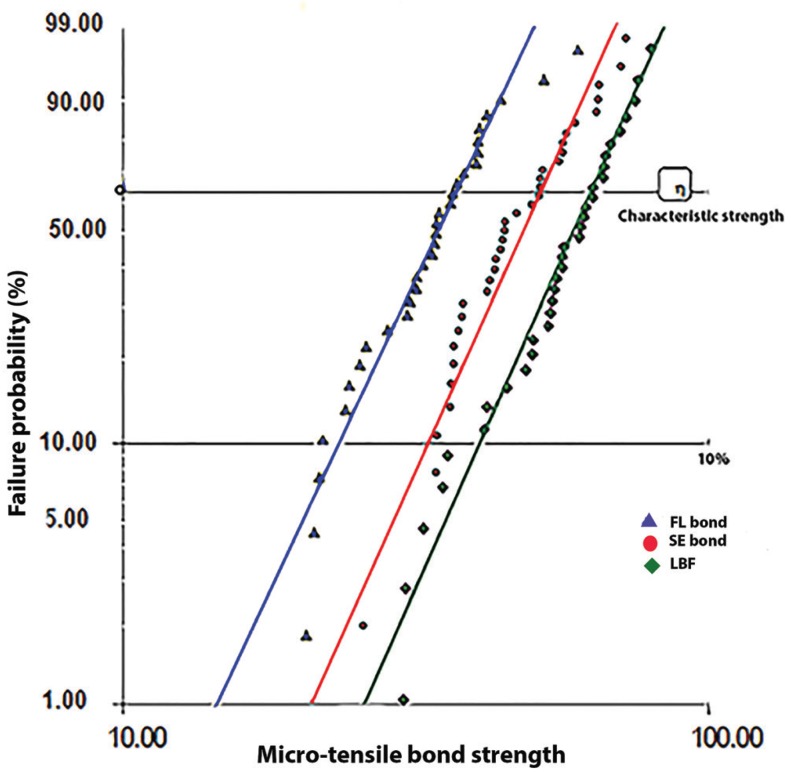
Weibull plot of the micro-tensile bond strength of experimental groups.

The obtained characteristic strength of groups ranged from 35.92 for the FL fluoride containing group to 56.49 for LBF fluoride containing dental adhesive, as shown in Table 2. The obtained Weibull modulus m revealed a similar trend in all experimental groups. However, the Weibull modulus was slightly lower for LBF group. The Weibull plots of the microtensile bond strength of the study groups are shown in Fig. 2.

**Table 2:**
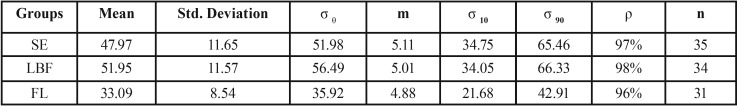
Micro-tensile bond strength and Weibul parameters of experimental groups.

**Figure 3:**
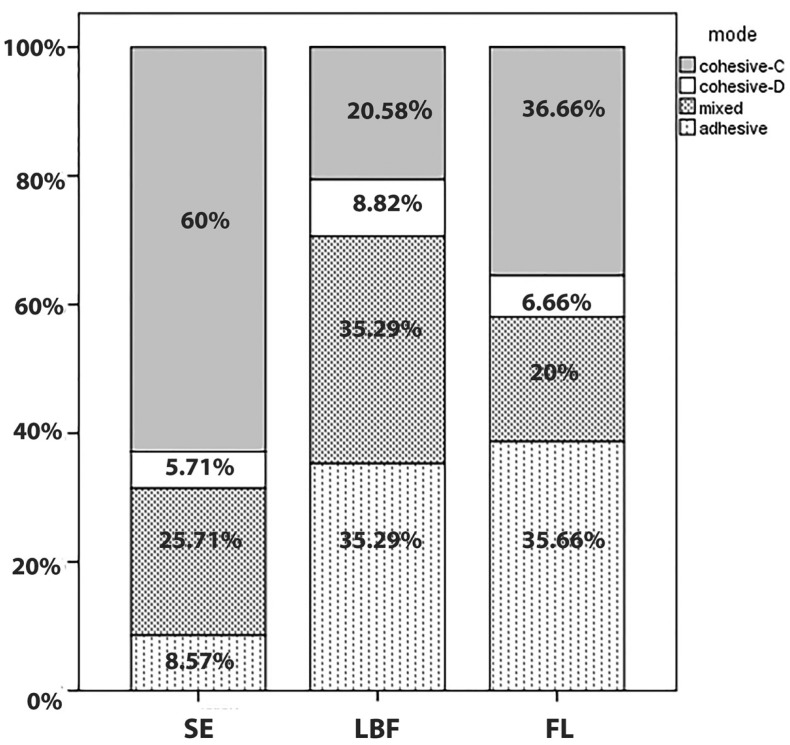
Distribution of failure modes (%) of each experimental group.

**Figure 4:**
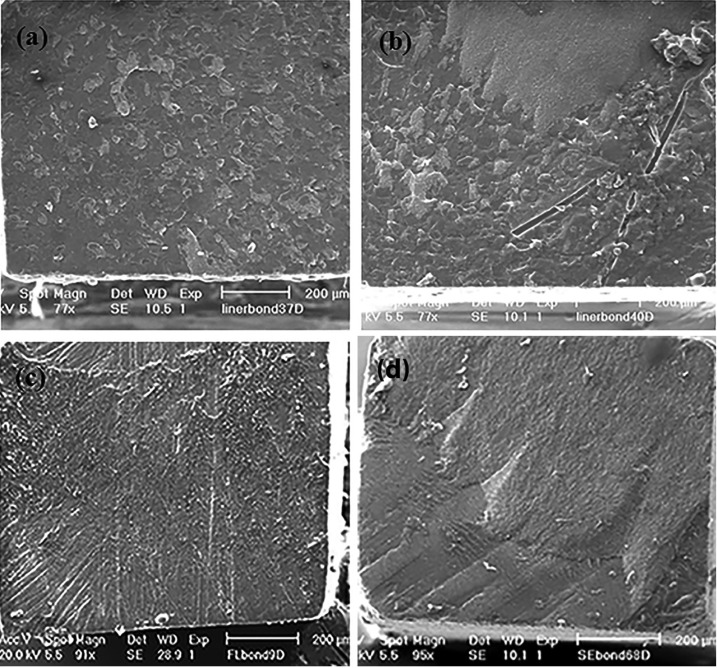
Representative scanning electron microscopy images of fractured surfaces.(a) adhesive failure in Liner Bond F (LBF) group, (b) Mixed failure pattern in Liner Bond F (LBF) group, (c) Adhesive failure in Fluorobond II (FL) (d) Cohesive failure pattern in SE bond.

Failure analysis using SEM revealed that the fracture pattern in most of the specimens of SE bond was cohesive into the bulk of resin composite (60%) (Fig. 3). On the other hand in fluoride containing dental adhesives most specimens showed adhesive or mixed failure pattern rather cohesive failure (Fig. 4) .

- Cross-sectional microhardness (CSMH)

The mean hardness values and standard deviations of experimental groups of dentin and enamel sections at baseline and after pH-cycling regime are described in  Table 3 and  Table 4.

**Table 3:**
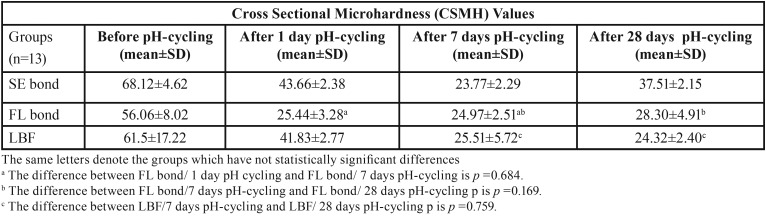
Mean dentine cross-sectional microhardness values (standard deviation) in each experimental groups.

**Table 4:**
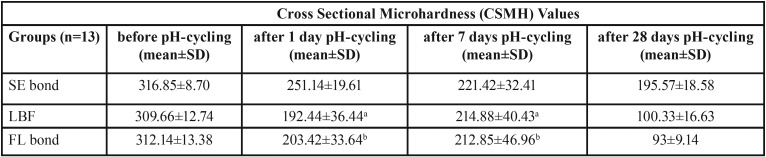
Mean enamel cross-sectional microhardness values (standard deviation) in each experimental groups.

The mean CSMH values and standard deviations of samples before pH-cycling procedure and after seven days and 28 days of pH-cycling at dentine and enamel are described in  Table 3 and  Table 4, respectively.

As shown, the time of pH-cycling significantly affected the hardness values of the dentine underneath the SE bond adhesive. In this regard, the CSMH values declined up to day 7. After that, the hardness values significantly increased at day 28 of the pH-cycling process (*p*≤0.001). On the first day of pH-cycling, the FL bond group also showed trend similar to SE bond group; and the mean hardness value significantly reduced after a day of pH-cycling (*p*≤0.001), however, this reduction was not statistically significant between the first and seventh days of pH-cycling (*p*=0.684). The CSMH values in FL bond samples increased after day 7 up to day 28 as SE bond group, although this difference was not statistically significant (*p*=0.169). The changes in hardness values of dentine in the LBF group showed a behaviour similar to that of the SE bond group up to the day 7 (*p*≤0.001). However, unlike the SE bond and LBF groups, the hardness value of dentin slightly decreased up to day 28 (*p*=0.759).

**Figure 5:**
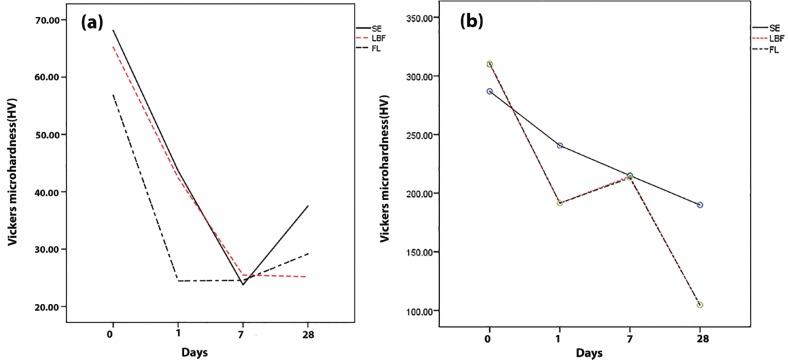
Profile of cross-sectional microhardness changes of experimental groups in (a) dentine and (b) enamel.

The time of pH-cycling process also had a significant effect on the enamel hardness underneath the self-etch adhesive systems. Accordingly, the enamel hardness values of all experimental groups decreased until day 28 of pH-cycling (*p*<0.05). However, a slight increase was observed between day 1 and day 7 in the FL bond and LBF groups (*p*=1.00). The profiles of changes in microhardness of enamel and dentine underneath the adhesive systems during the pH-cycling regime are shown in Fig. 5.

**Figure 6:**
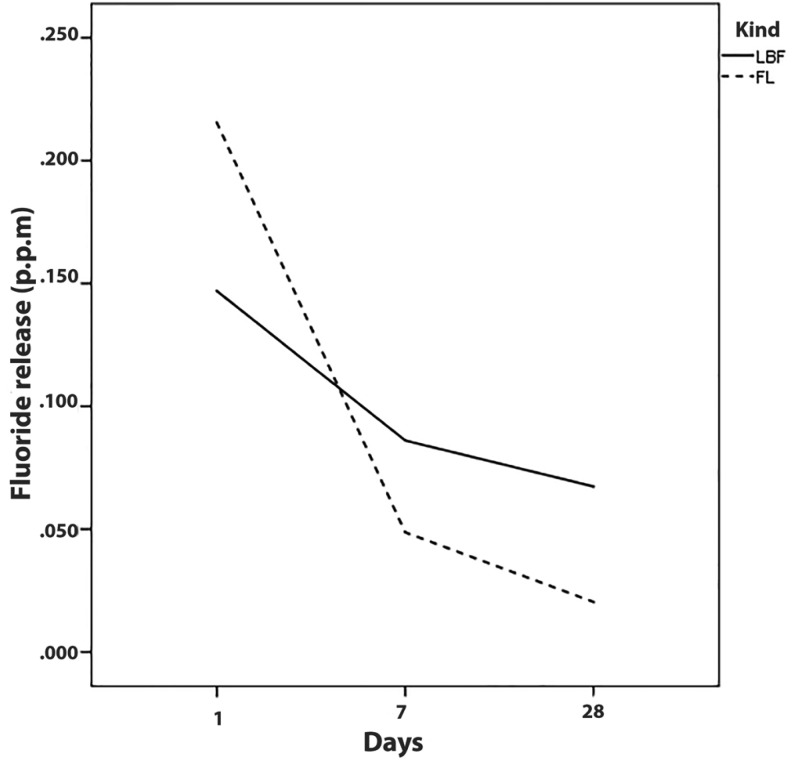
Profiles of fluoride release from each fluoride-containing dental adhesive during pH-cycling procedure.

- Fluoride release:

The profiles of fluoride release from each fluoride-containing dental adhesive at different experimental times of pH-cycling procedure are shown in Fig. 6. However, on the first day, the released fluoride from the FL bond (0.21±0.11 p.p.m) was greater than the LBF bond (0.14±0.065 p.p.m); it reduced dramatically until day 7 and also from day 7 until day 28 (*p*≤0.001). LBF adhesive also showed a trend similar to that of the FL bond; the released fluoride reduced from day 1 until day 28 (*p*≤0.001). However, the slope of the profile was blunter than the FL bond.

## Discussion

Many studies have shown the caries prevention effect of fluoride (16,17). Due to these findings, fluoride-containing restorative materials and adhesives have been developed to inhibit secondary caries formation. The potential benefits of these fluoridated materials include an effect on cariogenic bacteria by inhibition of metabolic enzymes (18), resistance to demineralisation and enhancement of remineralisation of adjacent tooth structures (19). It has to be considered that the caries prevention property of fluoride-containing products depends on the amount of released fluoride in the oral environment (4). In fluoride-releasing dental adhesives, it also notable that the released fluoride does not influence the bond strength of adhesive systems to tooth structure.

Accordingly, the aim of this study was to assess the fluoride release and dentine bond strength of self-etch fluoridated dental adhesives, and also the resistance to demineralisation of the dentine and enamel adjacent to these adhesive systems.

The results of the study showed significantly higher dentine bond strength in the LBF and SE groups compared to the FL group. A meta-analysis review of the performance of the ten most common dental adhesives presented the Clearfil SE Bond as the second best-performing adhesive (20). Clearfil SE Bond contains 10-methacryloyloxydecyl dihydrogen phosphate (MDP) (Table 1) in which an excellent chemical interaction with hydroxyapatite (HAp) has been clearly proved (21). The chemical interaction of 10-MDP with HAp of tooth structure can be ascribed to the formation of MDP-calcium salts on the surface of HAp crystals (21,22). Moreover, it is determined that longer interaction of MDP and hydroxyapatite leads to the precipitation of CaHPO4.2H2O on the top of MDP-calcium salts. Accordingly, the higher bond strength of SE and LBF groups compared to the FL group can be attributed to the presence of the 10-MDP monomer in their structure (22). Furthermore, in the current study the bond strength was evaluated after 3,000 cycles of thermo-cycling procedure. As described earlier, the longer interaction of 10-MDP with tooth mineral structure may form the sTable MDP-calcium salts. On the other hand, Fluorobond II contains 6-Methacryloxyhexyl 3-Phosphonoacetate (6-MHPA) monomer in its composition. Shakaya *et al.* (23) reported a significant reduction of 6-MHPA containing dental adhesives after 500 cycles of thermo-cycling. They suggested that this reduction is probably produced due to hydrolysis of dentine/adhesive interface and the water sensitivity of the chemical composition (23). Lida *et al.*, (24) also reported significantly lower bond strength for 6-MHPA containing dental adhesives compared to the 10-MDP containing dental adhesives (24). The highest dentine bond strength of LBF group obtained in this study can be justified by releasing and incorporation of water soluble sodium fluoride filler into the dentine just beneath the hybrid layer. Shinohara *et al.* (12), found a positive effect of released fluoride in fluoride-containing dental adhesives on the adhesive/dentine interface. They said that the fluoride might interact with the dentine beneath the hybrid layer, leading to the dentine remineralisation (12). Nakajima *et al.* (25) attributed the improved dentine bond strength in fluoride-containing dental adhesives after three and six months storage in water to the increase in less soluble fluoroapatite mineral concentration due to the slow release of fluoride into the dentine beneath the hybrid layer (25). Despite the high bond strength value of LBF group, we found a greater prevalence of adhesive and mixed failures in both fluoride-containing dental adhesives compared to the fluoride-free SE bond adhesive. This finding did not agree with the results of meta-analysis of Leloup *et al.* (26), who reported that the higher bond strength value caused the higher the rate of cohesive failure (26). As we observed, the highest prevalence of adhesive and mixed failure was in the LBF group (~70%). We speculated that the easily released fluoride from sodium fluoride filler of LBF may interfere with the integrity of dentine/adhesive interface. The FL group contains S-PRG filler, which is produced by pre-reacted glass ionomer (PRG) technology. These fillers are produced by the reaction between acid-base reaction between fluoroalominosilicate glass and polyalkenoic acid to form a wet hydrogel. It is determined that these fillers can provide a continuous fluoride release via a ligand exchange between the fluoride ions and cations within the pre-reacted hydrogel (27,28). Nevertheless, our study did not confirm sustained fluoride release of SPRG filler containing dental adhesive. In both fluoride containing dental adhesives, the fluoride release significantly declined between day one and day 7 as well as between day 7 and day 28. The results of current study showed the cross-sectional microhardness values dramatically reduced between the days of first and seventh in both dentine and enamel of all experimental adhesives which revealed that this low fluoride release does not any effect on resistance to demineralisation in tooth structure. Dionysopoulos *et al.* (29) also reported a low amount of fluoride ion released from fluoride-containing dental adhesives during the pH-cycling regime. They concluded that this low fluoride release did not influence the lesion formation of tooth enamel (29). Similar results have been reported by Peris *et al.* (30), who didn’t find a significant difference in the caries depth between fluoride-free and fluoride-containing dental adhesives. A very low fluoride release concentration was also reported in their study (mean concentration of 0.002ppm) (30). According these results and those of the current study, it seems that the low amount of fluoride released in fluoridated dental adhesives does not affect the resistance to demineralisation of either dentine or enamel. Contrary to our results, several studies have confirmed the influence of fluoride released from fluoride-containing dental adhesives on resistance to demineralisation and secondary caries formation in tooth structure (31,32). The reported controversial results reveal the importance of further studies to focus on the potential beneficial effect of added fillers in dental adhesives composition for remineralising and antimicrobial approaches.

## Conclusions

Within the limitations of the current study, it can be concluded that fluoride released from fluoride-containing dental adhesives did not affect the resistance to demineralisation of human tooth structure. Moreover, the released fluoride may adversely influence the integrity of resin/dentine interface and, hence, the bond strength and bond durability of adhesive system to dentine or enamel structure.
